# Martini 3 Coarse-Grained Force Field for Carbohydrates

**DOI:** 10.1021/acs.jctc.2c00757

**Published:** 2022-11-07

**Authors:** Fabian Grünewald, Mats H. Punt, Elizabeth E. Jefferys, Petteri A. Vainikka, Melanie König, Valtteri Virtanen, Travis A. Meyer, Weria Pezeshkian, Adam J. Gormley, Maarit Karonen, Mark S. P. Sansom, Paulo C. T. Souza, Siewert J. Marrink

**Affiliations:** † Groningen Biomolecular Sciences and Biotechnology Institute and Zernike Institute for Advanced Materials, University of Groningen, Groningen 9747 AG, The Netherlands; ‡ Department of Biochemistry, University of Oxford, South Parks Road, Oxford OX1 3QU, United Kingdom; § Natural Chemistry Research Group, Department of Chemistry, University of Turku, FI-20014 Turku, Finland; ∥ Molecular Microbiology and Structural Biochemistry, UMR 5086 CNRS and University of Lyon, Lyon 69367, France; ⊥ The Niels Bohr International Academy, Niels Bohr Institute, University of Copenhagen, Copenhagen 2100, Denmark; # Department of Biomedical Engineering, Rutgers, The State University of New Jersey, Piscataway, New Jersey 08854, United States

## Abstract

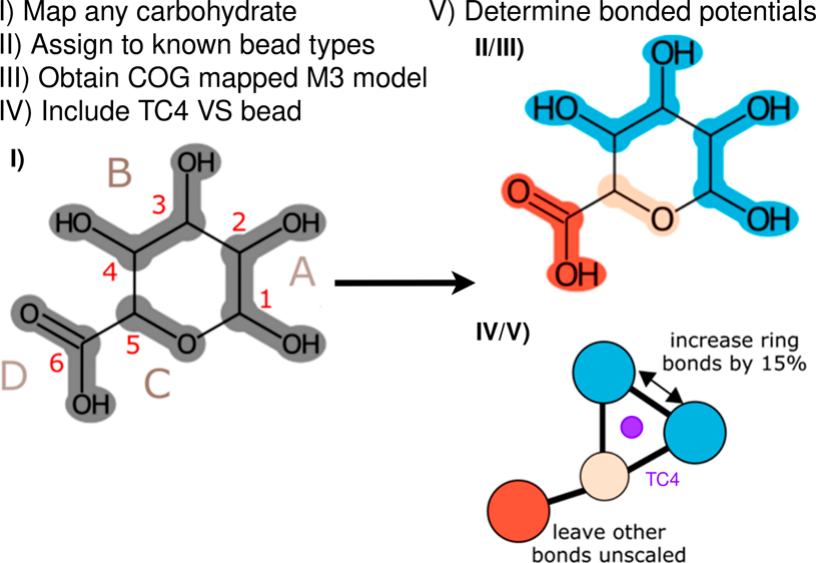

The Martini 3 force field is a full reparametrization of the Martini coarse-grained model for biomolecular simulations. Due to the improved interaction balance, it allows for a more accurate description of condensed phase systems. In the present work, we develop a consistent strategy to parametrize carbohydrate molecules accurately within the framework of Martini 3. In particular, we develop a canonical mapping scheme which decomposes arbitrarily large carbohydrates into a limited number of fragments. Bead types for these fragments have been assigned by matching physicochemical properties of mono- and disaccharides. In addition, guidelines for assigning bonds, angles, and dihedrals were developed. These guidelines enable a more accurate description of carbohydrate conformations than in the Martini 2 force field. We show that models obtained with this approach are able to accurately reproduce osmotic pressures of carbohydrate water solutions. Furthermore, we provide evidence that the model differentiates correctly the solubility of the polyglucoses dextran (water-soluble) and cellulose (water insoluble but soluble in ionic liquids). Finally, we demonstrate that the new building blocks can be applied to glycolipids. We show they are able to reproduce membrane properties and induce binding of peripheral membrane proteins. These test cases demonstrate the validity and transferability of our approach.

## 1 Introduction

Carbohydrates (sugars) are an important class of biomolecules. They play an active role in cell biology as they are, for example, part of the cell metabolism^1^ or signaling pathways.^2^ In addition, they are structural building blocks for many biomacromolecules such as polysaccharides, glycosylated proteins and lipids, and nucleotides. Furthermore, in research for sustainable materials, carbohydrates are a key factor as they can be obtained from renewable stock.^3^ Therefore, simulating these molecules in complex systems by molecular dynamics (MD) is of high interest to a wide audience of researchers. MD studies can give near atomistic resolution of processes impossible to capture with experimental techniques and therefore often complement experimental studies.

Due to the limits in spatiotemporal resolution of models representing all atoms explicitly, so-called coarse-grained (CG) models are often used in MD simulations. In CG models, several atoms are grouped into one effective interaction site. This greatly increases the simulation speed and reduces computational costs. Among the most popular CG models for (bio)molecular dynamics is the Martini model.^4,5^ The Martini model has been widely applied across many fields ranging from biomolecular science to material science.^6−9^ In the Martini model,^5^ about four heavy atoms are grouped into one interaction center, called bead. The interactions between beads represent the nature of the underlying chemical groups; the strength of the interaction is selected from a discrete set of LJ interactions by reproducing thermodynamic data - mostly the free energies of transfer between water and different organic solvents. In addition to the regular Martini beads, smaller bead sizes (S- and T-beads) are used for groups that are represented at higher resolution such as aliphatic or aromatic ring fragments.^5,10^ The speed-up of Martini over atomistic simulations is partially caused by the fact that dynamics at the CG level are faster than in atomistic simulations. However, that also means dynamic properties such as diffusion are more difficult to interpret and often require a scaling approach.

Within the framework of the previous version of the Martini model (i.e., version 2), several parameters for carbohydrates have been developed and successfully applied.^11−26^ However, Martini 2 has several pitfalls when it comes to parametrization of molecules, which lead to unphysical behavior.^27^ This was especially apparent for the carbohydrate model. As pointed out by several authors, carbohydrates in Martini 2 tend to largely overestimate the self-aggregation propensity.^12,13^ Although some of these problems could be alleviated by either increasing the interaction strength of carbohydrates with water^13^ or by replacing regular bead types with small beads,^18^ these solutions were *ad hoc* and did not resolve the underlying imbalance of the bead interactions. To overcome these deficiencies, the third edition of the Martini force field comprises a complete reparameterization of the original force field. Rebalancing of the nonbonded interaction, as well as extended verification against physicochemical reference data, led to an improved description of previously problematic molecular interactions.^5,10,28−31^

In the present work, we develop a consistent strategy to parametrize arbitrary carbohydrate molecules accurately within the framework of Martini 3, going beyond recently published models for specific carbohydrates.^32−34^ In particular, we develop a canonical mapping scheme that decomposes large carbohydrates into mono- and disaccharides, which are parametrized based on matching physicochemical reference properties and atomistic reference simulations. To facilitate application of this scheme, automatic mapping from all-atom simulations is implemented in the fast-forward program^35^ for all carbohydrate fragments considered. In addition, we propose guidelines for assigning bonds, angles, and dihedrals to allow for a more accurate description of carbohydrate conformations than in the Martini 2 force field. At the moment, bonded interactions for specific complex carbohydrates have to be mapped from an atomistic reference simulation unless the specific compounds are presented in this paper. Generic bonded parameters are subject to a forthcoming publication.

The remainder of this paper is organized as follows: First, we present the parametrization strategy of carbohydrates starting with monosaccharides (Section 2.1), subsequently extending to disaccharides and more complex carbohydrates (Section 2.2). Afterward, we validate the transferability of our approach by demonstrating that we can accurately model four example systems, previously impossible to consistently model with Martini 2, in particular, reproduction of osmotic pressures of monosaccharides (Section 3.1), solution and solubility behavior of two polysaccharides namely dextran and cellulose (Sections 3.2 and 3.3), and glycolipid mediated binding of peripheral membrane proteins (Section 3.4). Finally, we discuss limitations of our new carbohydrate modeling strategy and conclude.

## 2 Parametrization of Carbohydrates with Martini 3

We derived parameters for Martini 3 carbohydrates following the general rules for creating Martini models, as outlined in the main parametrization paper.^5^ However, we aimed at not only deriving the optimal parameters for the specific carbohydrates considered but also at casting them in a consistent framework as much as possible such that we obtain a generalized strategy for modeling arbitrary carbohydrates.

### 2.1 Monosaccharides

#### Scope

Carbohydrates as a molecule class display a large heterogeneity in size, structural connectivity, and isomerization states. Most biologically and technologically important carbohydrates are either hemiacetal monosaccharides or formed by condensation reactions of hemiacetal sugars. The hemiacetal monosaccharides exist mostly as six membered carbon rings (pyranoses) or five membered carbon rings (furanoses). These monosaccharides can undergo enantiomerization reactions switching between an open form and a ring-closed form in solution. However, the total fraction of ring-open structures is typically very low. Therefore, in this paper, we only consider closed-ring monosaccharides. Furthermore, the C1 carbon, which is called anomeric carbon, is chiral. This chirality of hemiacetal monosaccharides also causes a specific type of isomerization called anomerization. Depending on the position of the alcohol group connected to the anomeric carbon, a carbohydrate either has an α- (axial alcohol) or β- (equatorial alcohol) conformation. Within the Martini 3 carbohydrate model, we do not distinguish between the anomers in the case of monosaccharides. As shown in Table S1, the geometries (i.e., bond lengths) are so similar that we can treat these molecules as one class as was done in the previous Martini carbohydrate models.

#### Mapping

**Figure 1:**
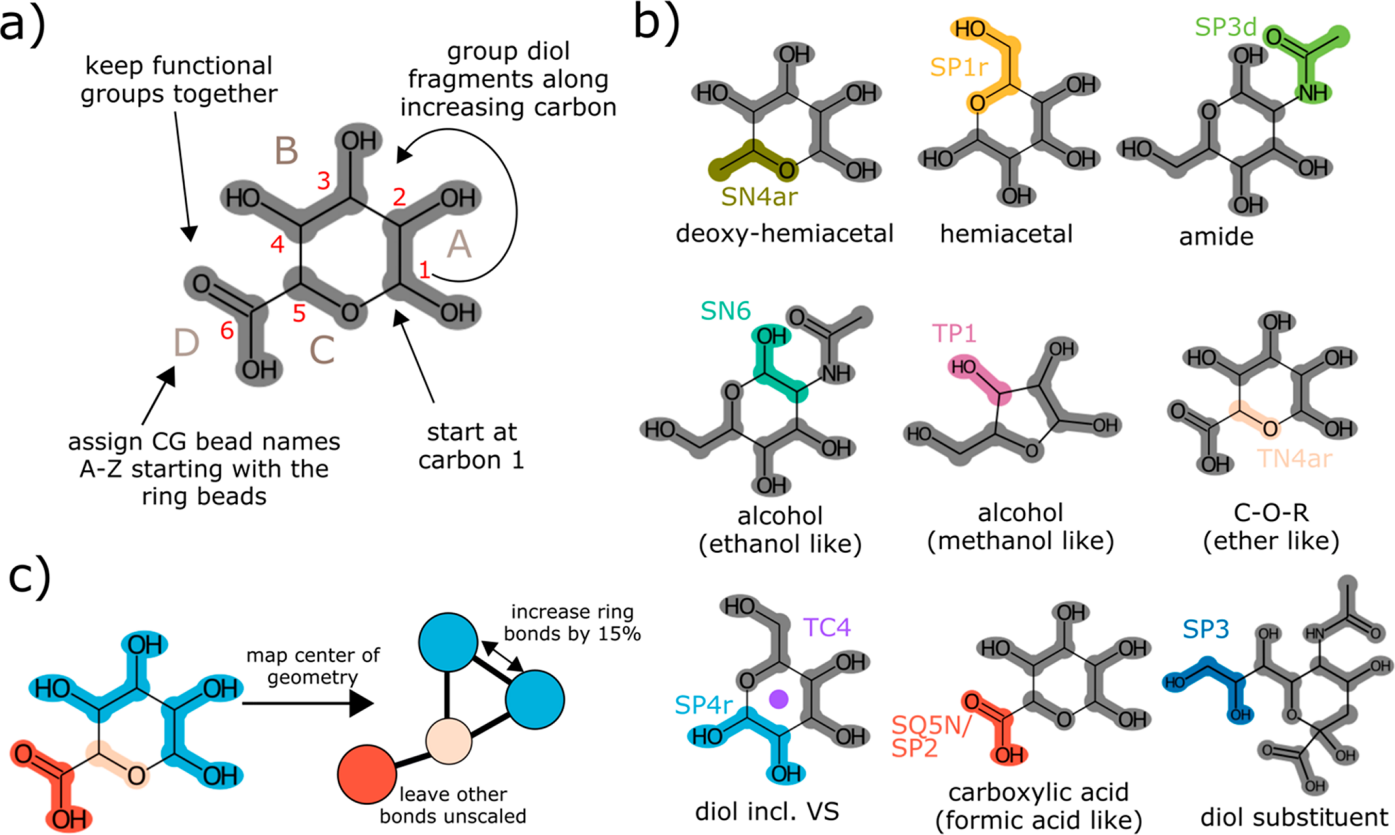
Parametrization strategy for monosaccharides. a) Systematic mapping scheme; b) Bead assignment for all fragments found in monosaccharides; c) Design principle for bonded interactions.

The mapping describes which atoms at the all-atom level are represented by a single bead in the CG model. The mapping choice determines all subsequent model choices and therefore requires careful consideration. In general, in the context of Martini 3, one maps 2–5 heavy atoms into one bead, where the number of mapped atoms and their connectivity determine the bead sizes. All carbohydrate mappings are derived obeying the following three base rules, which are aimed at making the mappings transferable and consistent across complex carbohydrates too (Figure 1a).

A mapping 1) maximizes the number of diols assigned to a single bead, therefore maximizing the number of 4:1 mapped atoms; 2) keeps functional groups together as much as possible; and 3) starts at the anomeric carbon and proceeds counterclockwise for grouping fragments. The first rule recognizes that the most commonly found fragments in sugars are diols and the hemiacetal group. Having as many similar fragments as possible simplifies the model and allows finding a good bead type for that fragment across the many test cases. The second rule is needed in cases where substituent groups are present and supersedes rule 1, if needed. For example, in d-glucuronic acid (Figure 1a), the acid substituent group is kept together, making the ring fragment smaller, and in the case of Neu5Ac, the three side chains are also kept together (Figure S1). Thus, the ring fragment becomes a 3–1 mapping. The third rule ensures that equivalent fragments are generated for the different sugars and makes a canonical naming scheme possible. To simplify handling and analysis of our model, we have developed a canonical naming scheme. The ring beads in carbohydrates are named A, B, and C, where the A-bead is the first bead and always includes the anomeric carbon according to the previously defined mapping direction and the C-bead always includes the ether oxygen. Substituents are named after letters in the alphabet in the order in which they are attached to the main ring beads. For example, in the case of glucuronic acid, we have one substituent named D.

#### Bonded Interactions

**Figure 2:**
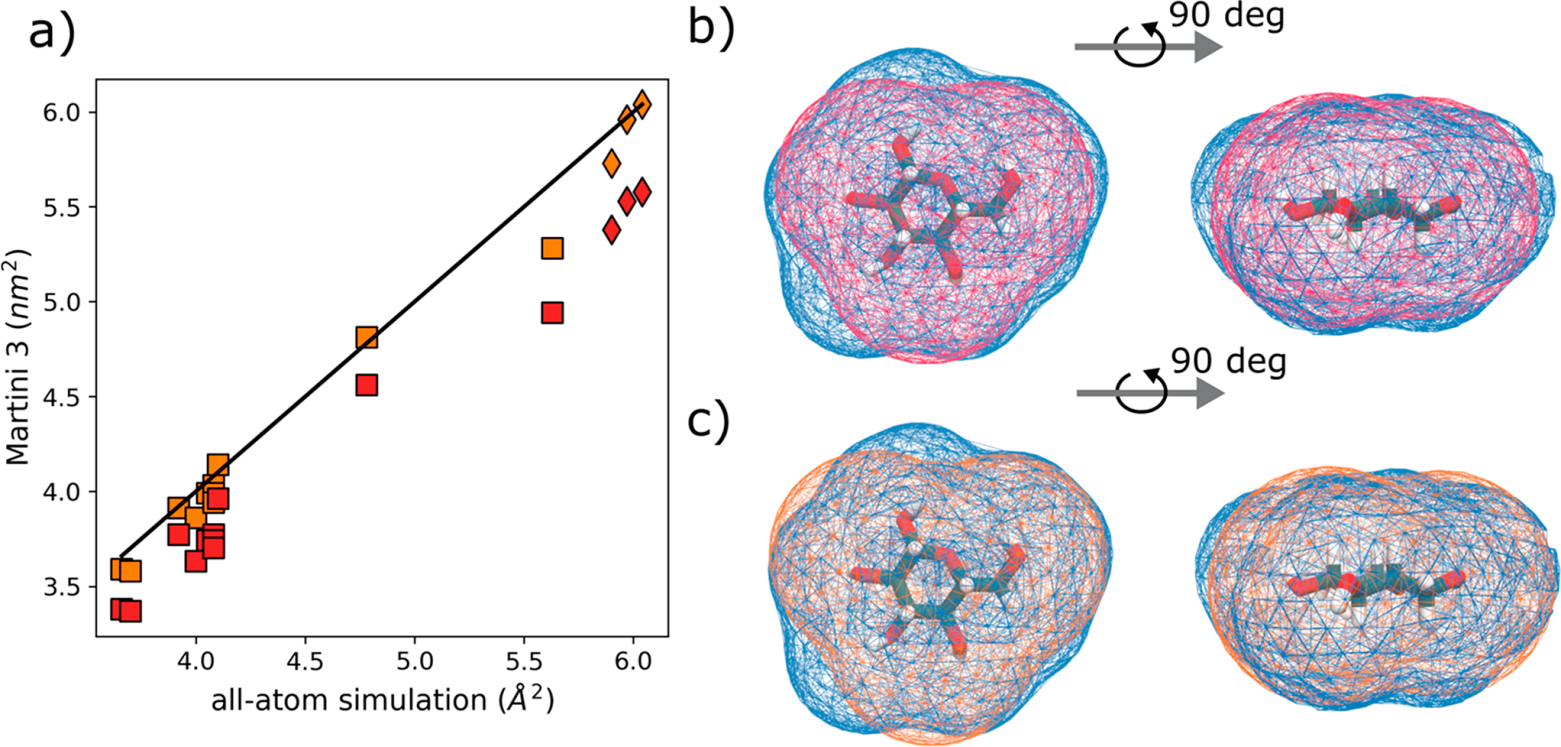
Molecular shape optimization. a) Solvent accessible surface area (SASA) compared between atomistic reference simulations and Martini 3 with unscaled bonds (red) and scaled bonds (orange), for monosaccharides (squares) and disaccharides (diamonds); b) Connolly surfaces for glucose comparing atomistic (blue) to Martini 3 (red) before scaling the bonds; c) Connolly surface for glucose after scaling the bonds comparing atomistic (blue) to Martini 3 (orange).

As the monosaccharides are rigid triangles at the CG level, we decided to model them using constraints. The constraint length was derived by mapping the center of geometry (COG) of the atomistic reference structure to the CG representation following the previously derived mapping scheme. One of the key problems of sugars in the old Martini 2 model was the overestimated aggregation propensity.^12,13,27^ As analyzed by Alessandri and co-workers, this is partially caused by too short bond lengths and a poor representation of the molecular volume at the CG level.^27^ Thus, in Martini 3 it is recommended to match the molecular volume of the atomistic structure as closely as possible.^27^ To assess the molecular volume, we conducted atomistic simulations of 11 monosaccharides and 3 disaccharides at the all-atom level using the most popular force fields for sugars (i.e., Glycam06h,^36^ Charmm36,^37^ and GROMOS^38^). Based on these simulations, the SASA was computed as outlined in the Methods section and compared to CG SASA values. As the SASA depends only on the bead size, we assigned bead types of an appropriate size, based on the mapping guidelines of the Martini 3 model. As most of the fragments are 4:1 mapped, displaying branched moieties or 3:1 mapped linear groups, we considered mostly S-beads in accordance with the Martini 3 mapping guidelines. For some of the remaining 2:1 mapped fragments, the appropriate class of T-beads was used instead. Figure 2a shows the comparison of the Martini 3 SASA values against atomistic simulation data. The models, which are obtained by simply mapping the center of geometry (red symbols), underestimate the molecular volume significantly. Overall deviations for the unscaled model are of the order of ∼8%. Thus, it stands to reason that this approach leads to similar problems as observed in Martini 2.^27^ To further elucidate the problem, we computed the Connolly surfaces^39^ of the molecules involved. An example is shown for glucose in Figure 2b-c. One can clearly see that the unscaled coarse-grained surface (blue) does not match the atomistic reference surface (red). To improve the agreement with the molecular volume, we followed the approach suggested by Alessandri et al.^27^ and increased the bond lengths of the beads, forming the sugar rings. Such a scaling approach has also been used previously to improve interactions of PIP lipids, which contain a carbohydrate headgroup.^30^ To keep our model transferable to carbohydrates not considered, we explored a compound independent scaling factor. A uniform scaling of 15% over the COG mapped distances was found to greatly improve the agreement of the SASA (orange data points Figure 2a) and at the same time be applicable to all monosaccharides. Also, the Connolly surfaces show a better agreement (Figure 2c). Now the orange and the blue surface align well for most parts of the molecule.

#### Bead Choices

Nonbonded interactions are assigned from a discrete set of interaction levels (referred to as bead type) by selecting those types that optimally reproduce the available physicochemical reference data. In this particular study, we selected the bead types by matching the free energies of transfer from octanol to water for 11 monosaccharides. We note that only experimental values for glucose are available^40−42^ (−17.52 ± 1). Thus, we set out to measure the remaining partition coefficients experimentally ourselves.

The value obtained for glucose (−17.81 ± 0.5) matches the previously published values well, giving confidence in the choice of experimental method. Table S2 summarizes the experimentally determined partition coefficients. The bead assignments were then optimized under the constraint that the same fragments must have the same bead type to be consistent with the building block approach of Martini. For example, inositol consists of three diol units. Thus, all beads in inositol must have the same type, and it fixes the choice for the diol bead fragment.

**Figure 3:**
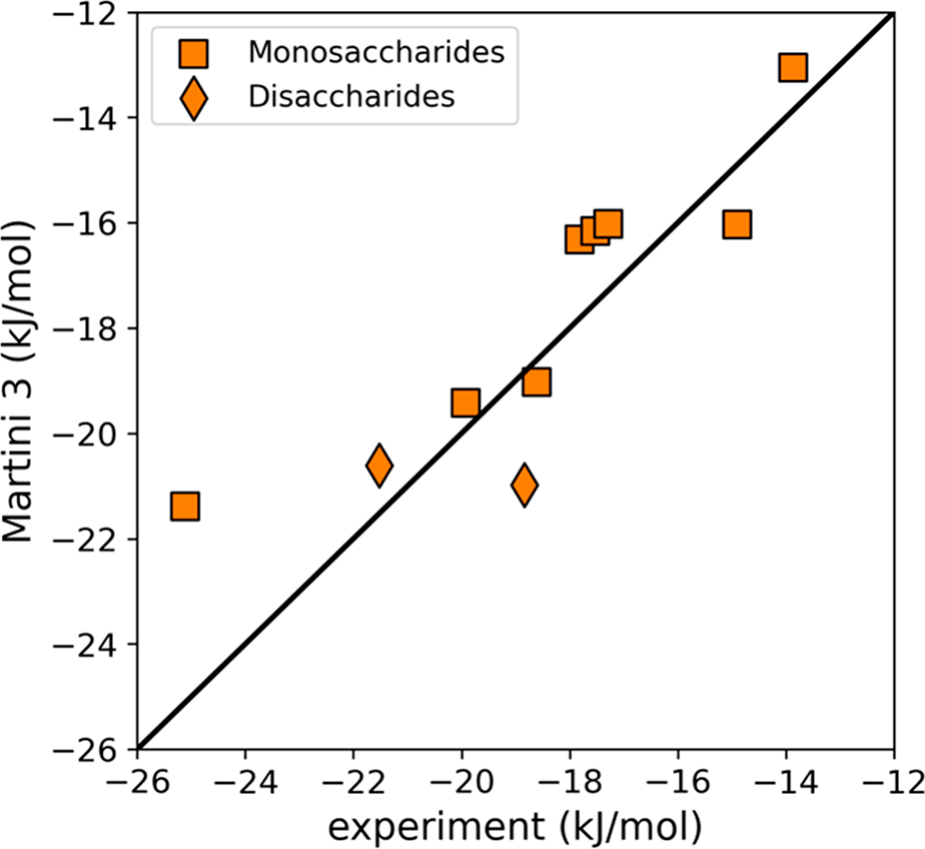
Free energies of transfer. Octanol-water free energies of transfer were computed using the Martini 3 model developed here and compared to either newly experimentally measured or existing literature values for monosaccharides (squares) and disaccharides (diamonds). See Table S2 for actual data. The error for all points was less than 0.4 kJ/mol.

Starting with that assignment, the choices for the other fragments could be optimized. Figure 1b shows the final bead assignments for the monosaccharides considered. Figure 3 shows the correlation of the experimental versus coarse-grained free energies of transfer. We note that the mean absolute error across all monosaccharides is only 1.5 kJ/mol which we consider excellent. For comparison, it is about the same as the average error in transfer free energy for the small molecules considered in the Martini 3 parametrization, which is 2.0 kJ/mol.^5^

In addition to assigning a bead type for each fragment in the three membered ring, our model also contains a virtual interaction site (VS), which is placed at the center of geometry of the ring. The VS is a massless interaction site that has the bead type TC4 across all monosaccharides. Note that all bead assignments were done with the TC4 interaction site present. This VS helps to reproduce interactions with aromatic groups - through so-called ring stacking^43−45^ - which in Martini needs to be captured through a hydrophobicity component to the interaction. In addition, the extra TC4 bead counteracts the mismatch in number of non-hydrogen atoms per bead, which results from the use of S-beads to represent the four non-hydrogen ring atoms. As described in the Martini 3 guidelines, the maximum mismatch should be one non-hydrogen for each ten non-hydrogen atoms mapped by CG beads.^5^ Such an approach has already been used successfully in parametrization of phosphatidylinositide lipids with Martini 3.^30^ In the Supporting Information, we assess the effectiveness of the virtual site by computing the potential of mean force (PMF) profiles between indol and glucose (Figure S2).

### 2.2 Disaccharides and More Complex Carbohydrates

#### Mapping

**Figure 4:**
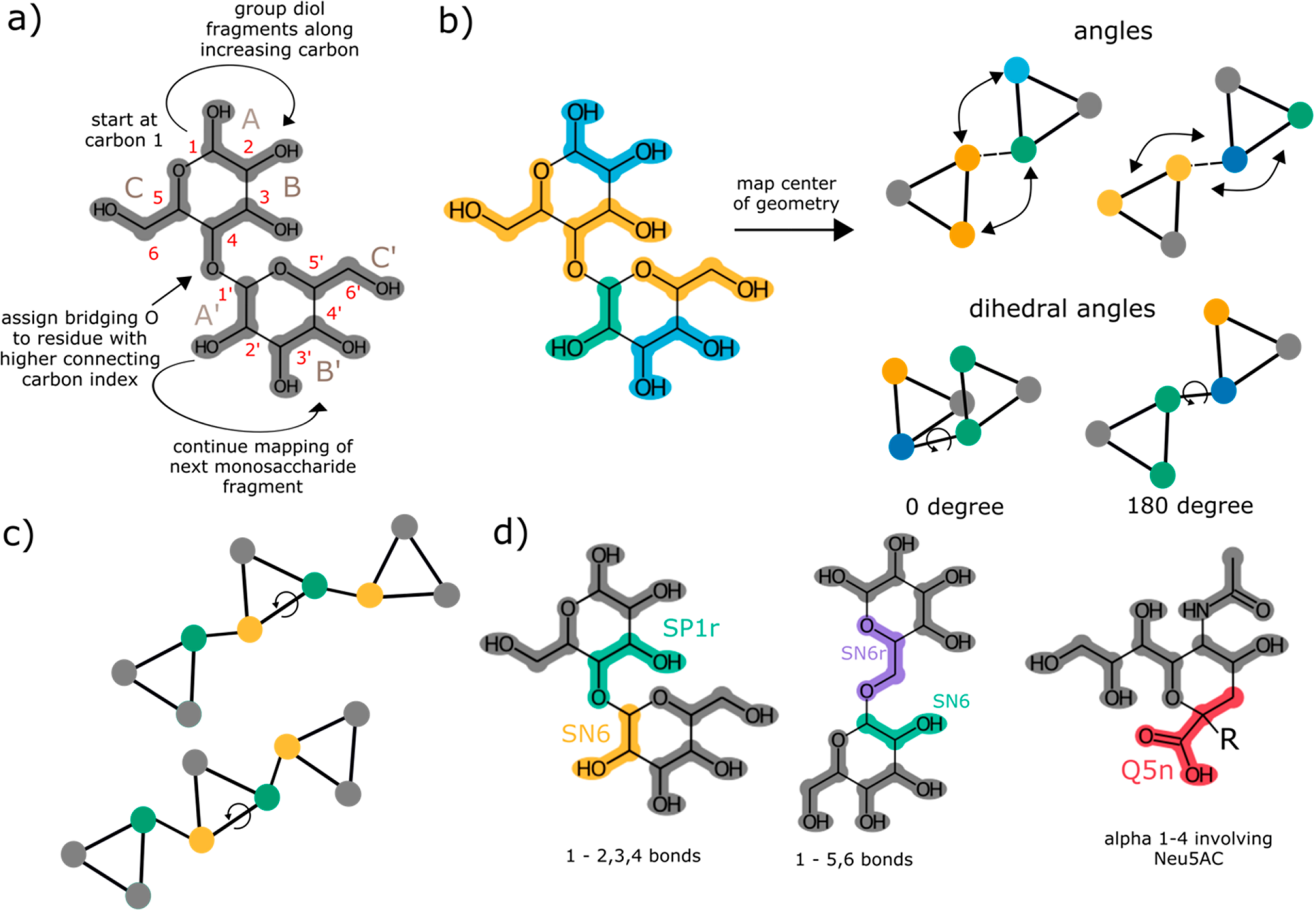
Parametrization strategy for oligo- and polysaccharides. a) Systematic mapping strategy for complex carbohydrates; b) Angles and dihedrals introduced between two linked monosaccharide fragments; c) Dihedral angle introduced between three consecutive monosaccharide fragments; d) Bead assignment for all fragments not found in monosaccharides.

The mapping of disaccharides directly follows from the mapping of the monosaccharides, that is, each constituting monosaccharide is mapped following the rules outlined above. The only thing to consider is the problem of where to separate the disaccharide into its constituting monomers. We adopt the same approach as previously used by the CHARMM-GUI glycan reader,^46^ considering the connecting atoms to belong to the monosaccharide with the higher bead number at the CG level. For example, in the case of lactose (C_12_H_22_O_11_), which is an α-1,4 linkage of glucose and galactose (see Figure 4a), the connection is between the B and A beads of the CG model and between carbon 5 and 1 of the atomistic model. We consider the ether fragment to belong to the B-bead fragment. This mapping allows to obtain transferable mappings between all disaccharides, and since it is consistent with the CharmmGUI convention, it allows automatic forward and backward mapping using already existing tools such as fast-forward^35^ or backward.^47^ The complex carbohydrates and polysaccharides are mapped following the same principle, which also holds for branched carbohydrates such as the GM1 lipid. This scheme also allows Martini 3 carbohydrates to keep a building block approach, which was a concern in the previous Martini 2 model, where the CG geometry of the sugar rings needed to change from monosaccharide (triangular topology) to oligosaccharide models (linear).^48^

#### Bonded Interactions

Bonded interactions are derived following the same strategy as for the monosaccharides, that is, mapping and matching the underlying atomistic reference distributions. To optimally represent the conformational space underlying carbohydrate oligomers and polymers, we define all angles spanning the bond between two monosaccharide repeat units as well as one dihedral controlling the rotation around the glycosidic bond (Figure 4b). In case more than two monosaccharide repeat units are connected, an additional dihedral angle is introduced which spans three repeat units (Figure 4c).

This dihedral angle defines the relative orientation of the n and n+2 residue with respect to the plane spanned by the n+1 residue. We notice that this dihedral is particularly important especially for longer carbohydrates, as it relates to the stiffness of the polysaccharides. Polysaccharides formed by condensation can either have α- or β-based bonds. We noticed that the difference in bond length at the CG level is significant between an α- or β-bond due to the relative positioning of the two rings with respect to each other. Therefore, in our model, we distinguish explicitly between the two anomers when found in a poly- or oligosaccharide. In the case of the dextran polymer to allow for better matching of the underlying AA distributions and improved stability, we use three-bonded neighbor exclusions. However, for all other models, only the one-bonded neighbors are excluded as is standard in Martini lipids. Furthermore, for angles that are covered by any dihedral potential, the restricted bending potential introduced by Bulacu et al.^49^ is used to reduce instabilities from angles becoming colinear. As for the monosaccharides, we have assessed how well our model represents the molecular volume by computing SASA values for three disaccharides: lactose, sucrose, and trehalose (Figure 2a). The correlation with the atomistic SASA values is equally good as for the monosaccharide case, given the bond scaling is retained. However, it should be noted that the connecting bonds between two monosaccharide repeat units are left unscaled. This minimal number of bonded interactions provides a good representation of the all-atom conformations and also leads to a numerically stable model. Moreover, it improves in selectivity and functionality compared to the Martini 2 carbohydrates, which treated glycosidic bonds indiscriminately and even had problems modeling flexible α-1,6 linkages.^11^ Note that bonded parameters for other complex carbohydrates (especially dihedrals) should be mapped from an atomistic reference simulation. Using generic bonded interaction to combine arbitrary carbohydrates is beyond the scope of the current paper and will be discussed in a forthcoming publication.

#### Bead Choices

Bead types of the fragments which are equivalent in both mono- and disaccharides are retained following the building block spirit of Martini. Therefore, the only bead types which need to be defined are those involved in the glycosidic bond. As shown in Figure 4d, hexose bonds can be collected into two groups based on the newly generated fragments. One group contains the 1–1, 1–2, 1–3, and 1–4 bonds. The appropriate bead type of this fragment, SP1r, is directly taken from the monosaccharides (cf. Figure 1b). However, we further validated this choice by reproducing the free energies of transfer between octanol and water of the disaccharide’s trehalose and sucrose. Deviations for both compounds were acceptable with errors of 0.5 kJ/mol for trehalose and ∼3.0 kJ/mol for sucrose. The other group contains 1–5 and 1–6 glycosidic bonds, in which case another bead type needs to be assigned.

The new bead is similar to the hemiacetal fragment but for the change of one OH-group to an ether group. Such a change will likely result into less strong self-interactions, which is captured by the SN6r bead type having one level less strong self-interaction. Finally, in the case of N-acetylated neuraminic acid attached via a 1–4 bond (Figure 4d last panel), we group the carboxylic acid together with the remaining carbon fragment in order to avoid a 2:1 fragment being generated with a short bond length. As a consequence, a new large bead is used instead of the two smaller beads. The bead type was determined to be the standard carboxylic acid bead from the original Martini 3 publication. We note that as a result of the generalized mapping scheme, as well as the fact that biologically relevant carbohydrates are confined to certain linkages, with this small number of new bead types almost all biologically relevant sugars can be constructed.

## 3 Validation

In order to demonstrate the transferability of the model and assess if the model improves on the multiple issues of the Martini 2 model, we analyzed a number of test cases, considering four different target systems.

### 3.1 Osmotic Pressures

**Figure 5:**
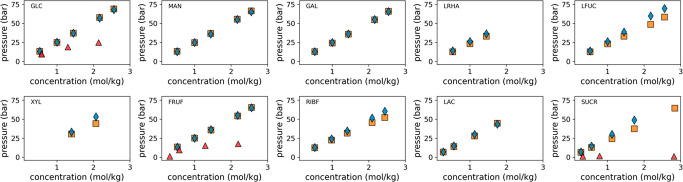
Osmotic pressure of carbohydrate solutions. Osmotic pressure for ten different carbohydrates measured from simulations using the presented Martini 3 model (orange) and the regular Martini 2 model (red) in comparison to experimental data (blue diamonds) collected from various sources.^50−53^ Mono- and disaccharide codes are indicated with each panel: GLC = d-glucose, MAN = d-mannose, GAL = d-galactose, LRHA = l-rhamnose, LFUC = l-fucose, XYL = d-xylose, FRUF = d-fructose, RIBF = d-ribose, LAC = lactose, SUCR = sucrose. The error on CG osmotic pressures was less than 1 bar and thus smaller than the symbols shown.

The major drawback of the Martini 2 carbohydrate model is the overestimation of the aggregation propensity.^12,13,27^ To quantitatively assess the aggregation propensity of solute molecules in solution, the osmotic pressure is frequently computed as a function of the concentration. An osmotic pressure lower than experiment is indicative of too strong an aggregation propensity. This procedure has been applied to reparametrize both CG and AA force fields for carbohydrates and other molecules.^13,37,54,55^ To compute the osmotic pressure for our carbohydrates, we have adopted the procedure originally proposed by Luo and Roux.^56^ The molal concentrations were determined from the box density after preparing it at a certain molar concentration. Since experimental measurements are generally reported in molal units, we considered this approach to be more accurate at higher concentrations. Figure 5 shows the osmotic pressures for eight monosaccharides and two disaccharides in the concentration range from 0 to 2.5 molal. The scaled bond model (orange squares) shows an excellent agreement with the experimental data (blue diamonds) in the lower concentration range (<1.5 molal) across all carbohydrates. This already presents a significant improvement over the Martini 2 model for which data was available for only three carbohydrates (red triangles).

For the higher concentration range (>1.5 molal), we see that our model follows the overall trends well but shows some deviations, in particular, for ribose, sucrose, fucose, and xylose. The lower pressures observed for these carbohydrates suggest some remnants of the stickiness problem to be still present. However, overestimated solute–solute interactions in concentrated solutions are not unique to CG force fields like Martini. Even popular atomistic force fields such as CHARMM36 or GLYCAM06 have been shown to significantly underestimate osmotic pressures and therefore exaggerate aggregation in simulation of carbohydrates in water. Agreement with experimental data is especially bad at higher concentrations (>2.5 molal).^37,54,58^ The reported deviations for these atomistic force fields are similar or even much worse than the deviations observed for our CG carbohydrate model.

Keeping this in mind, we conclude that our model reproduces the osmotic pressure very well overall and constitutes a significant improvement over the Martini 2 carbohydrate models. In addition, we note that the accuracy of our model is comparable to default atomistic force fields. Thus, we consider the match to be good enough.

### 3.2 Solution Properties of Dextran

**Figure 6:**
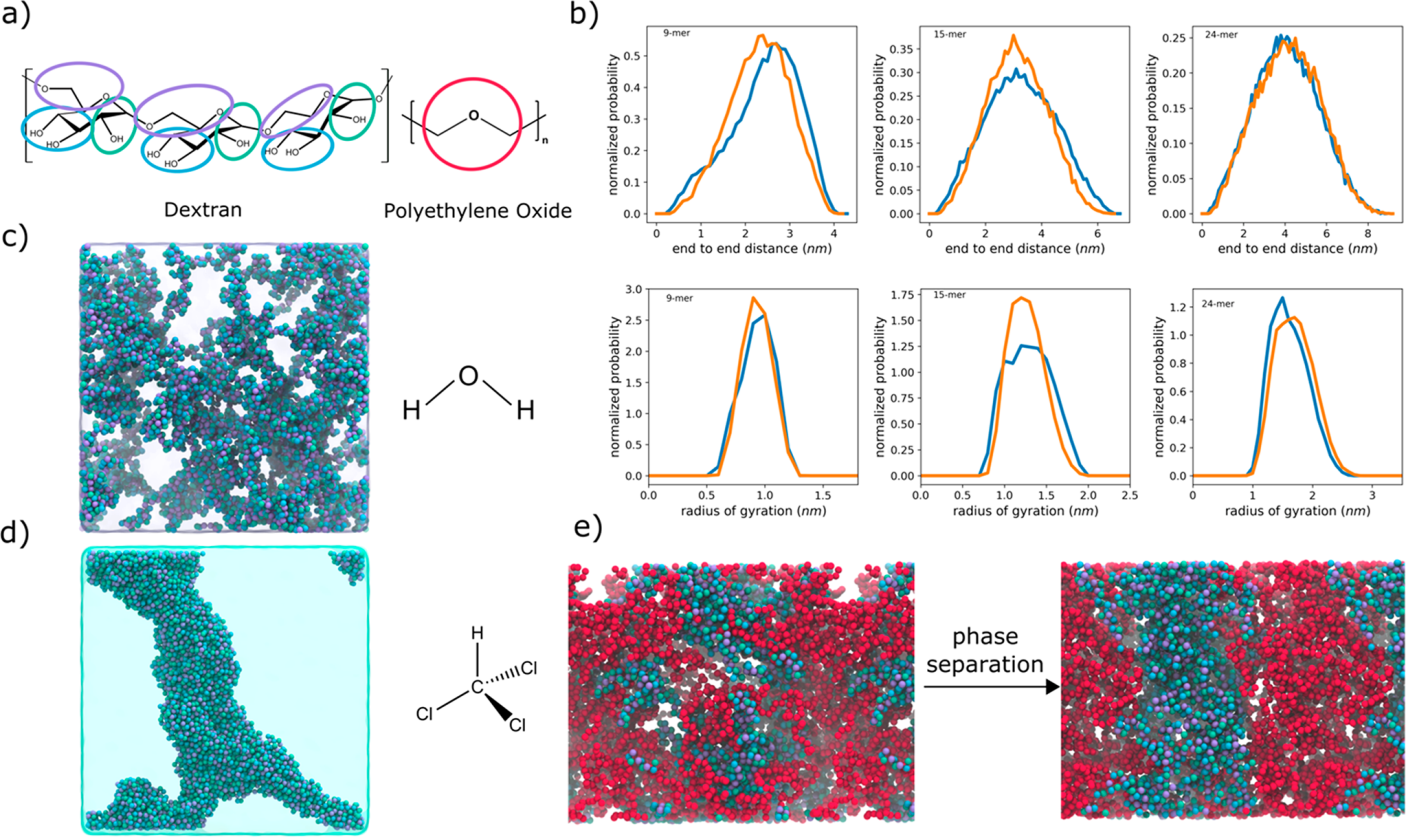
Solution properties of dextran. a) Mapping of dextran and poly(ethylene oxide) (PEO) at the Martini level. The colors of dextran correspond to the bead types as found in Figures 1 and 4. b) Radius of gyration and end-to-end distance of dextran oligomers with 9, 15, and 24 repeat units from all-atom CHARMM36m^46,57^ simulations (blue) and the new Martini 3 model (orange). c) Snapshot of the aqueous solution of dextran (50 repeat units). d) Dextran globule formed in chloroform, a nonsolvent, starting from dispersed polymers. e) Aqueous solution of dextran (65 repeat units) and PEO (180 repeat units) at the beginning of the simulation in the fully mixed initial state (left) and after 3 μs of simulation (right). The solvent is omitted for clarity.

Dextran is a branched polysaccharide, consisting of α-1,6 glucose units with α-1,3 connected glucose units branching off it.^59,60^ Unlike other polyglucoses - such as cellulose or amylose - dextran is fully water-soluble even at high molecular weight fractions (Figure S3). Here, we investigate the solution properties of dextran in water to demonstrate that our model is capable of reproducing not only properties of mono- and disaccharides but also properties of complex polysaccharide solutions. Whereas high molecular weight dextran is usually highly branched, lower molecular weights (with degrees of polymerization below 100) typically display only in the order of 5% or less branching.^59,60^ Since we mostly utilize such lower molecular weights, all dextran used here is linear and has no branches. Dextran bead types (Figure 6a) were assigned based on the previously presented concepts, and the bonded parameters were obtained by matching atomistic CHARMM36m^46,57^ simulations. Subsequently, we investigated the dilute solution conformations of three oligomers differing in the degree of polymerization (DoP 9, 15, 24). Figure 6b shows distributions of the radius of gyration as well as the end-to-end distance for the all-atom model (blue) and the Martini 3 model (orange). We note that the agreement is excellent between both sets of simulations for both metrics. The end-to-end distance is typically related to polymer stiffness via the persistence length. Since our end-to-end distance distributions agree well with the AA model, our persistence length of the dextran oligomers will be very close to the persistence length of the AA model. In contrast, the scaling of the radius of gyration is related to the dilute solution thermodynamics via for example Flory theory.^61^ Being able to match the AA radius of gyration distributions closely indicates that we capture the solution thermodynamics well, suggesting that our model has a good balance between self- and water interactions.

We continued to investigate further, if dextran is still soluble at higher weight fractions. Figure 6c shows a snapshot of a dextran (DoP 50) solution at 10 w/w%, after 5 μs of simulation. Clearly no phase separation or strong aggregation is visible. In the Supporting Information (Figure S4), we further show the radial distribution functions (RDFs) for the polymer–polymer and polymer–water interactions. Both indicate that the dextran is fully solubilized in water. To put these results into perspective, we also simulated dextran in chloroform for 5 μs. Chloroform was experimentally determined to be a nonsolvent (Figure S3). Already within a few hundred nanoseconds, the polymers all aggregate into a periodic cluster (Figure 6d). Note that the concentration is the same in both simulations. The RDF for the polymer and polymer chloroform interactions (Figure S4) also shows the increased aggregation and depleted solvent interaction. We conclude our dextran model is fully water-soluble and insoluble in chloroform, as expected for these concentrations and molecular weights.

Our final test case involves an aqueous system of dextran and poly(ethylene oxide) (PEO). Dextran is known to phase separate from PEO in a ternary mixture within water forming an aqueous two-phase system (ATPS) via liquid–liquid phase separation (LLPS).^62,63^ ATPSs are important both in biomedical applications,^64^ for instance, microfluidic separation, and in biological research,^62^ where they are used as compartmentalizing cytosol mimetics. Martini 3 has previously been shown to be able to capture LLPS of biomimetic compounds,^65^ and our model should thus also be capable of simulating such systems accurately. To this end, we generated a mixed PEO (DoP 180)-dextran (DoP 65) system using polyply.^66^ We note that the used molecular weights and concentrations have previously been reported to form an ATPS.^62^Figure 6e shows the system at the start and end of a 2 μs simulation. Clearly the system has phase separated from the initial mixed state into a dextran rich phase and a phase enriched in PEO. Analysis of density profiles along the Cartesian *z*-axis supports this conclusion (Figure S5). Overall, we showed that our dextran model matches solution conformations of atomistic simulations well, reproduces solubility trends in water and chloroform, and forms an ATPS with PEO. All these observations demonstrate the validity of this carbohydrate polymer model.

### 3.3 Solution Properties of Cellulose

**Figure 7:**
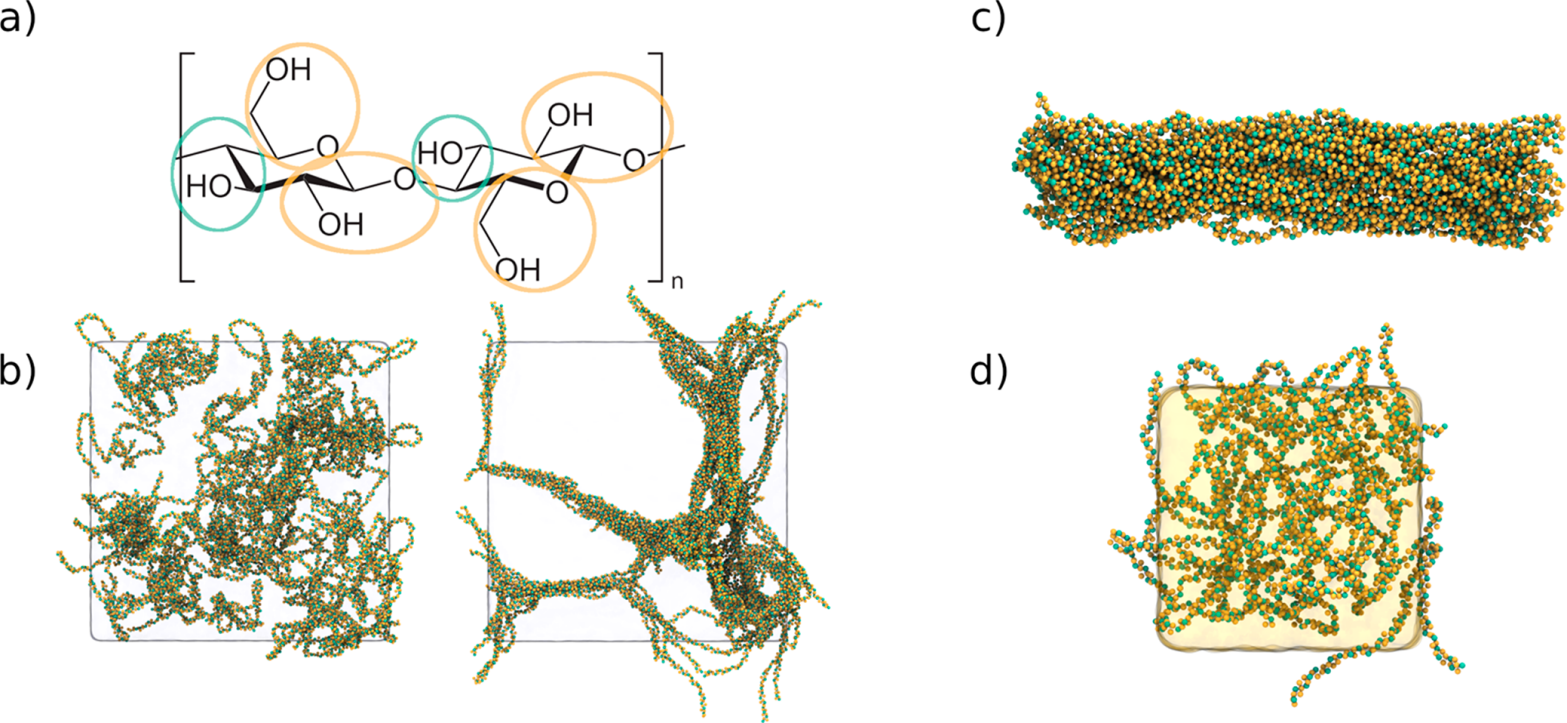
Solution properties of cellulose. a) Mapping of cellulose at the Martini level. The colors correspond to the bead-types as found in Figure 1. b) Cellulose solvated in water. The figure on the left shows 100 glucose chains with a degree of polymerization of 50, placed randomly in water. The figure on the right shows the state of the system after 2 μs of simulation. c) A cellulose Iβ fibril (36 chains with a degree of polymerization of 50) after a 2 μs simulation, solvated in water. d) Cellulose chains after a 2 μs simulation, being solvated in the [BMIM][Cl] ionic liquid.

In the previous two sections, we have shown that our Martini model produces carbohydrate molecules, which are water-soluble and do not suffer from the same aggregation effects as seen for Martini 2. However, not all carbohydrates are water-soluble. For example, cellulose is famously known to be insoluble in water. In order to verify that our balance of interactions is reasonable and does not favor water-soluble systems too much, we assessed solution properties of short cellulose analogs. In particular, we simulated poly(β-1,4)-glucoses with a DoP of 50 (Figure 7a). At these lengths, it is known to form stable crystals in water that do not solubilize.^67^ We started by building a system in a perfectly mixed state and random chain conformations and simulated it for 3 μs. Figure 7b shows the starting configurations as well as the last frame of the simulation. Clearly the cellulose analog starts aggregating, even forming small fiber-like structures. We further investigated if preconstructed crystals of cellulose are stable. To this end, a cellulose crystal (1β) was built using cellulose-builder,^68^ solvated, and simulated for 2 μs. Reassuringly, the fibril remains stable and insoluble in water, although we do observe the structure deviating from the original forward-mapped crystal structure. Whereas cellulose is insoluble in water, it does dissolve in some ionic liquids (IL). As Martini 3 is also capable of simulating ILs,^69^ we proceeded to investigate what happens if the solvent of the above systems is changed from water to [BMIM][Cl], which cellulose is known to dissolve.^70^Figure 7d shows the system after 2 μs of simulation. In contrast to the simulation with water, we do not observe a fiber-like structure being formed. In addition, RDFs (Figure S6) show that the cellulose remains fully solvated. This test case demonstrates that our Martini 3 model is in principle capable of investigating cellulose solubility.

### 3.4 Binding of Peripheral Membrane Proteins to Glycolipids

**Figure 8:**
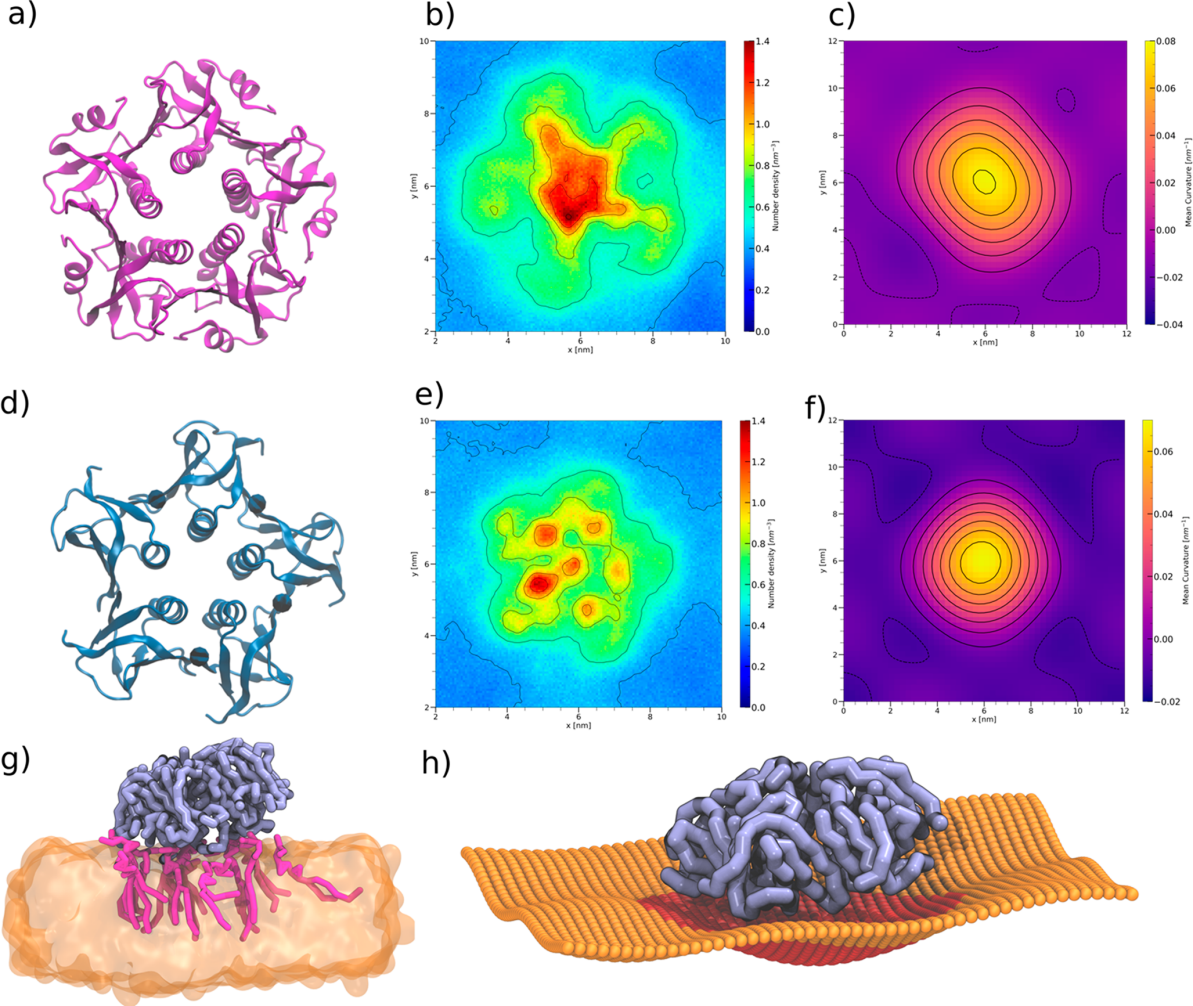
Binding of peripheral membrane proteins to glycolipids. a) Rendering of the cholera toxin subunit B (CTxB) protein structure (PDB 3CHB); b) 2D lipid density map of GM3 around CTxB; c) 2D curvature plot of the membrane around CTxB; d) Rendering of the Shiga toxin subunit B (STxB) protein structure (PDB 2C5C); e) 2D lipid density map of Gb3 around STxB with equivalent binding sites indicated by 1–3; f) 2D curvature plot of the membrane around STxB; g) CTxB (violet) bound to the membrane with GM3 lipids shown in pink and the rest of the membrane shown as transparent; h) projected membrane surface with the CTxB protein structure in the center.

Lipid and lipid–protein interactions in complex membranes are one of the main application areas of Martini.^71−73^ However, glycolipids, which consist of a carbohydrate headgroup and various lipid tails, suffered from the same problem of excess aggregation as other sugar molecules.^18^ To show that our model is transferable to lipids and proteins, we study the interaction of peripheral membrane proteins with glycolipids. In particular, we focus on bacterial Shiga and cholera toxins, secreted by *Shigella dysenteriae* and *Vibrio cholerae*, respectively. Both toxins are associated with several human diseases, e.g., diarrhea.^34^ In addition, these toxins are of special interest for their applications in biophysical experiments, targeted drug delivery, and cancer therapy.^74−76^ Both toxins are AB_5_ proteins, composed of an enzymatic active A and membrane binding homopentameric B subunit. The B subunit of cholera toxin (CTxB; Figure 8a) and Shiga toxin (STxB; Figure 8d) initiates the toxin internalization through binding to their natural receptors on the targeted cell plasma membrane: the glycolipid globotriaosylceramide (Gb3) for STxB and ganglioside (GM) for CTxB. Parameters for Gb3 and GM3 (monosialodihexosyl-ganglioside) have been designed using the presented strategy for parametrizing the carbohydrate headgroup. Lipid tail parameters were the same as in the default Martini 3 force field with adjusted linker mapping as explained in the Supporting Information, where the complete mapping for both lipids is shown (Figure S7).

First, we studied cluster formation of the GM3 lipids in a 10% POPC bilayer. Figure S8 shows the cluster-size distribution from the all-atom CHARMM36m simulation, an improved Martini 2 model proposed by Gu and co-workers,^18^ as well as the present model. In the AA simulation, GM3 mostly exists as monomers with dimers being much less likely. Higher order clustering is almost nonexistent. The same trends are captured by the fixed Martini 2 model, as well as our newly proposed Martini 3 model. We conclude that both Martini models perform equivalently well but slightly overestimate aggregation in relation to the AA simulations. However, we consider this a satisfactory result for a generic coarse-grained model. We note that the lipid tail parameters in Martini 3 are currently subject to further optimization.

Whereas realistic lipid mixtures in complex membranes were already possible to capture with the optimized Martini 2 models, binding of peripheral membrane proteins to their natural glycolipid receptor remained problematic.

The Martini 2 carbohydrate model shows no specific binding sites of Gb3 to STxB. In previous studies using Martini 2, Gb3 lipids were therefore tethered to the protein via a covalent bond based on the atomistic reference structure.^77^

To study the process of STxB binding to Gb3 lipids with our new Martini 3 model, we simulated the system under the same conditions as the previous Martini 2 study.^77^ To this end, a single STxB was placed above but not in contact with a POPC membrane, containing a 10% mole fraction of Gb3. During the simulation, we observe that the protein stably binds to the membrane, not leaving during the 10 μs long simulation. A control simulation without Gb3 lipids showed no binding of STxB to the membrane over the entire course of 10 μs. Similar to Shiga toxin, Cholera toxin also binds to glycolipid receptors but in this case GM lipids including GM3. Hence, we have simulated the binding of CTxB to a POPC membrane with 10% GM3 lipids, analogously to the STxB case. Also, CTxB spontaneously binds to the membrane within a few hundreds of nanoseconds and stays bound for the remaining 10 μs. We further analyzed binding of the glycolipids to the proteins by computing a 2D lipid density map around the centered proteins. The density maps (Figure 8b, Figure 8e) show an enrichment of glycolipids under the proteins in specific spots. For CTxB, we see the most dominant binding site to be in the center of the protein and weaker binding sites in the peripheries. To our knowledge, binding sites of GM3 have not been resolved for CTxB. In contrast, for Gb3 binding to STxB, three distinct binding sites per monomer have been resolved experimentally by X-ray diffraction.^78^ Two binding sites are found on the peripheries close to each other, and a third binding site is found at the bottom of the α-helix. In order to investigate to what extent our lipids bind in similar spots, we have computed the site-specific RDFs (see Figure S9) between the sugar parts of the glycolipids and those residues experimentally identified in binding. We see an increased probability to find a carbohydrate around each binding site, indicating that binding locations appear reasonable.

Finally, we have assessed the membrane curvature induced by binding of the two proteins. Both are known to induce curvature, which is an essential step in their endocytosis.^79−81^Figure 8c and Figure 8f show the 2D mean curvature under each protein, clearly demonstrating that our model can capture this behavior qualitatively. Previously, the curvature for STxB bound to Gb3 has been computed from atomistic simulations (0.034 ± 0.004 nm^–1^).^80^ The value obtained from the CG simulations is in the same ballpark (0.0260 ± 0.0001 nm^–1^). The level of membrane curvature induced by binding of CTxB is further illustrated in Figure 8h.

## 4 Discussion and Conclusion

In the present paper, we developed a consistent strategy to parametrize arbitrary carbohydrates with the Martini 3 force field. In particular, we presented a canonical mapping scheme that decomposes arbitrarily large carbohydrates into a limited number of fragments. Bead types for these fragments have been assigned by matching atomistic volumes and free energies of transfer from water to octanol. The best bead assignment yields a mean absolute error of about 1.3 kJ/mol compared to the experimental reference portioning data. In addition to the bead type assignment for fragments, guidelines for assigning bonds, angles, and dihedrals have been presented. These guidelines allow for a more accurate description of carbohydrate conformations than in the Martini 2 force field and can easily be expanded to more complex carbohydrates. We showed that models obtained with this parametrization strategy are able to reproduce osmotic pressures of carbohydrate water solutions to very good accuracy. Furthermore, we demonstrated that the model differentiates correctly the solubility of the poly glucoses dextran (water-soluble) and cellulose (water insoluble). Given that the difference between both models is only a single bead type and different bonded interactions, it speaks for the accuracy of our model being able to capture their differences. In the final test case, we illustrate that the model is applicable to glycolipids by showing that the clustering of GM3 is in good agreement with all-atom reference simulations. As a final validation, we analyzed the binding of peripheral membrane proteins Shiga and Cholera toxin to two glycolipid receptors. Here, we found that both proteins bind to the glycolipids and induce membrane curvature as expected. Taken together, these test cases demonstrate the validity and transferability of our approach.

However, some limitations apply as well. The osmotic pressure for certain monosaccharides indicated a too high self-interaction in the high concentration regime (conc. > 1.5 molal). Therefore, simulations concerning highly concentrated solutions need to be verified carefully. Furthermore, we note that while inclusion of the TC4 virtual site greatly helps in interactions with aromatic moieties, they remain lower than observed in all-atom models. This is especially true for conformations where a stacked interaction is enforced. For example, protein binding could be influenced by this effect. Whereas part of it is an intrinsic limitation of a CG model with fewer degrees of freedom, further improvement can be obtained by future improvements in the protein model or even careful revision of the Martini 3 interaction matrix - these are ongoing processes. We conclude that the rules for constructing carbohydrate models within Martini 3 lead to CG models that greatly improve in accuracy over Martini 2 and, at least in some aspects, are comparable to standard atomistic force fields employed in the field.

## 5 Methods

### 5.1 Experimental Measurements of the Partition Coefficients

Measurements were performed following a similar methodology as outlined in Virtanen et al.^82^ Measurements were done with an UPLC-DAD-HESI-Orbitrap-MS instrument. The column in the UPLC was an Aquity BEH Phenyl (100 × 2.1 mm i.d., 1.7 μm), and the mobile phase consisted of acetonitrile (A) and 0.1% aqueous formic acid (B). The elution gradient was carried out with a constant flow rate of 0.65 mL/min as follows: 0–0.1 min: 3% A; 0.1–3.0 min: 3.0–45.0% A (linear gradient); 3.0–3.1 min: 45.0–90.0% A (linear gradient); 3.1–4.0 min: 90% A; 4.0–4.1 min: 90.0–3.0% A (linear gradient); 4.1–4.2 min: 3.0% A. The ionization mode (negative/positive) of the mass spectrometer that was used for each compound depended on their ionization efficiency in either negative or positive mode; the one where each compound ionized more effectively in the test samples was then used for quantitative measurements. All measurements were done in triplicate, and quantitation for each compound was done from extracted ion chromatograms (EICs) from full scan MS analysis with a specific *m*/*z*-range for each compound. Integrated EIC areas were converted to concentrations before partition coefficient calculations with a calibration series done with a dilution series of each compound. Both the calibration series samples and the actual K_ow_ samples mass responses (integrated EIC areas) were normalized with an external standards mass response so that the possible variation in the mass spectrometers performance during the measurements and on different days could be taken into account.

### 5.2 Experimental Measurements of Dextran Solubility

0.5 g of dextran (15–20 kDa MW, Polysciences, Inc., USA, Cat# 01341) was added to a 20 mL scintillation vial. Afterward, 4.5 g of two solvents (Ultrapure H_2_O, and Chloroform) was then added, and mixtures were vortexed briefly for 10 s. Vials were then allowed to sit and equilibrate at 22 °C for 1 h before solubility as assessed via visual inspection.

### 5.3 All-Atom MD Simulations

All atomistic simulations were performed with GROMACS (2018.8 or 2021.5).^83^

#### Simulations of Mono- and Disaccharides

Carbohydrates were simulated using the GLYCAM06^36^ force field, the CHARMM36m^46,84^ force field, and the GROMOS54a7^38,85^ force field as outlined in Table S3. For each simulation, a single carbohydrate was solvated in a box of water with size 2.4 × 2.4 × 2.4 nm^3^ and simulated after short equilibration in the isobaric-isochoric ensemble at 1 bar. Temperatures were fixed at 310 K, 303.15 K, or 298.15 K for each respective force field. Each simulation was run for at least 200 ns using the default leapfrog integrator. All bonds were restrained with the LINCS algorithm.^86^ The Glycam06 and CHARMM36 simulations used TIP3P^87^ as a water model, whereas the GROMOS ones used the SPC model.^88^ For all force fields, the GROMACS specific recommended run settings were used. itp files for the carbohydrates were obtained from Glycam-Web and converted to GROMACS with acpype,^89,90^ or the CharmmGUI,^46^ or the automated topology builder (ATB).^38,85^

#### Simulations of Dextran Oligomers

Single chains of dextran oligomers in water were simulated using the CHARMM36m force field.^57^ Parameters and coordinates for three different degrees of polymerization (9, 14, 24) were obtained from the CHARMM-GUI.^46,91^ After equilibration, each simulation was run under constant temperature at 298.15 K using the v-rescale^92^ temperature coupling (τ = 1 ps) with a coupling group for solvent and polymer. Pressure was kept constant at 1 bar using the Parrinello–Rahman pressure coupling algorithm (τ = 5 ps, β = 4.5 × 10^–5^ bar^–1^). The simulations for the three oligomers were run for 6 μs, 3 μs, and 3 μs, respectively. Radius of gyration and the end-to-end distance were obtained from the simulation using the “gmx polystat” tool. Distributions were subsequently computed after discarding an equilibration time.

#### Simulations of Lipid Bilayers

All atomistic resolution lipid bilayer simulations used the CHARMM36m force field.^57^ The bilayers consisted of POPC as a major component and 10% glycolipids either GM3 or Gb3. Parameters and coordinates were obtained from the CHARMM-GUI.^46,91^ After equilibration, each simulation was run under constant temperature at 310 K using the Nose-Hoover temperature coupling (τ = 1 ps) with a coupling group for solvent and membrane. Pressure was kept constant at 1 bar using the semiisotropic Parrinello–Rahman pressure coupling algorithm in an *xyz* direction (τ = 5 ps, β = 4.5 × 10^–5^ bar^–1^). The simulations were run for 2 μs. Clustering of GM3 lipids was analyzed after mapping the trajectories to CG resolution with fast_forward.^35^ Subsequently using the “gmx clustsize” tool, lipids were counted as being in the same cluster, if the distance between the linker beads was less than 1.4 nm.

#### SASA Calculations

The solvent accessible surface area was computed using the double cubic lattice method by Eisenhaber et al. as implemented in the GROMACS software suite (i.e., gmx sasa).^93^ Instead of using the default VdW-radii for this calculation, the more recent VdW-radii proposed by Rowland and Taylor were used for the atomistic simulations.^94^ For the Martini simulations, the Vdw-radii were taken to be the minimum of the LJ self-interaction, which leads to three radii for the regular (0.264 nm), small (0.230 nm), and tiny (0.191 nm) beads. The probe size for both atomistic and CG simulations was 0.191 nm, and the SASA was averaged over at least 200 ns both for atomistic simulations and CG Martini simulations.

### 5.4 Coarse-Grained MD Simulations

CG simulations were performed with the Martini 3 force field^5^ or Martini 2 force field.^4^ Each Martini 3 simulation followed the standard simulation settings as outlined in the main publication,^5^ and the Martini 2 simulations followed the parameters as outlined by de Jong et al.,^95^ unless specified otherwise. The velocity rescaling thermostat^92^ (τ = 1 ps) and Parrinello–Rahman barostat^96^ (τ = 12 ps, β = 4.5 × 10^–5^ bar^–1^) were used to maintain temperature and pressure in production simulations. GROMACS version 2021.5 was used for all simulations unless otherwise stated. Bonds within monosaccharides were constrained with the LINCS algorithm.^86^

#### Free Energies of Transfer

All simulations pertaining free energies of transfer were carried out with the GROMACS software (version 2021.5),^83^ using the stochastic dynamics integrator^97^ (with inverse friction constant 1.0 ps^–1^) and a time step of 20 fs. Free energies of transfer of the carbohydrates were calculated as differences between free energies of solvation in water and octanol. Solvation free energies were computed by alchemical free energy transformations as implemented in the GROMACS package. All systems for the solvation free energy in water consisted of a single carbohydrate solute molecule and 1023 Martini water beads. The systems for the octanol solvation free energies consisted of 920 octanol molecules and 80 water beads representing a saturated octanol composition.

The calculations used in total 19 nonequally spaced windows, switching only the LJ interactions as the Martini molecules considered have no partial charges. Soft-core LJ potentials were applied following the recommended values.^98^ Each window was run under NpT conditions for 12 ns at 1 bar pressure maintained (τ = 4 ps). Temperature was maintained at 298.15 K. The derivative of the potential energy was recorded every 10 steps. All free energies of the transformation were estimated using the Bennetts Acceptance Ratio (BAR) method as implemented in the “gmx bar” tool. The error reported with the calculations is the statistical error estimate. The intramolecular interactions were not switched off for both sets of simulations.

#### Osmotic Pressure Calculations

The osmotic pressure was computed from simulations adopting the protocol originally proposed by Luo and Roux.^56^ A rectangular box was created in which the solute molecules were confined in the *z*-direction by a flat-bottomed potential to the center of the box. At a distance of 2.52078 nm from the center of the box, a harmonic force with a force constant 1000 kJ/nm^2^ was applied to the solute molecules. The box dimensions were taken to be 10.08312 nm in z-dimensions and 5.04156 nm in *x* and *y*. Previous to each run, a random configuration of solute and solvent molecules was created with polyply^66^ placing solute molecules only in the center of the box and the solvent in the entire box. After energy minimization, this setup was subjected to a 10 ns equilibration using a Berendsen barostat.^99^ Production simulations were run for 500 ns as previously used for atomistic simulation^55^ at a pressure of 1 bar. The temperature matched the temperatures reported with the experimental data sets. Following Sauter and Grafmuller,^54,58^ the pressure was coupled only in z-dimensions. The osmotic pressure was computed from the trajectory by recalculating the total force exerted by the solute particles onto the flat-bottomed potential averaged over the two potentials. Subsequently that force is divided by the *xy* area of the box. The ensemble average as well as an error were computed from the time-series of the osmotic pressure.

#### Simulation of Dextran Systems

Initial structures were built using polyply^66^ and subsequently subjected to an energy minimization. For the scaling simulations of the oligomers, first a short relaxation using the Berendsen barostat was run. Afterward they were sampled for 3 μs using the v-rescale barostat (6 ps, β = 4.5 × 10^–5^ bar^–1^).^100^ Mixing of PEO and dextran was studied in the same fashion; however, simulations were run for 5 μs. RDFs were computed using the “gmx rdf” tool. PEO parameters were taken from the polyply^66^ library (v1.3.0). The polymer–polymer RDF was computed as an average of RDFs for each polymer separately with the other polymers as to remove the correlation induced simply by the fact that neighboring repeat units are covalently bound to each other.

#### Simulation of Cellulose Systems

Starting configurations for the mixed state simulations in water and the [BMIM][Cl] ionic liquid were generated by placing glucose chains with a DoP of 50 randomly in a large simulation volume with the “gmx insert-molecules” tool and then solvating with the appropriate solvent. The Martini 3 IL parameters as published earlier were used.^5,69^ The starting structure of the cellulose fibril was created with the Cellulose Builder^68^ after which it was solvated in the same way as the previous systems. All systems were equilibrated for 50 ns at a temperature of 310 K with the system pressure controlled using the Berendsen barostat. Production simulations were run for 2 μs in the same temperature, using the Parrinello–Rahman barostat with isotropic coupling (τ = 12 ps, β = 4.5 × 10^–5^ bar^–1^).

#### Simulation of Peripheral Membrane Protein Binding

Initial structures of the lipid bilayers were built using TS2CG^77^ or obtained from the atomistic simulation by mapping the bilayer and resolvating it. All simulations were subjected to an energy minimization and equilibration. Subsequently all simulations were run under semiisotropic pressure coupling at 1 bar at 310 K temperature for 10 μs. Protein itp files were obtained using the martinize2 code as available on GitHub. The clustering of the GM3 lipids was analyzed following the same protocol as used previously.^18^ Membrane curvature was analyzed as described previously.^80^ 2D density maps were computed with “gmx densmap”. Both properties were computed as time-average over the last 7 μs. Specific binding sites were analyzed using ‘gmx rdf’.

## Notes

Mono- and disaccharide parameters developed in this paper were deposited in a GitHub repository (https://github.com/marrink-lab/martini-forcefields). Dextran and cellulose parameters are available from the polyply force field library, starting from polyply version >1.4.0. See the following link for more details: https://github.com/marrink-lab/polyply_1.0.

## Supporting Information Available

Detailed overviews pertaining to methodology, additional quantification (such as RDFs and partial densities of dextran, RDFs of cellulose and Gb3/Shiga toxin), insight into exact coarse-grained mapping, and information on reference partitioning coefficients (PDF)
