# Supplementary material for: Martini 3 Coarse-Grained Force Field for Carbohydrates
Source: J Chem Theory Comput. 2022 Nov 7;18(12):7555–69. doi: 10.1021/acs.jctc.2c00757 (PMC9753587; doi:10.1021/acs.jctc.2c00757)
Supplement: Supplementary file 1 — ct2c00757_si_001.pdf [file ct2c00757_si_001.pdf]

Supporting Information
Martini 3 Coarse-Grained Force Field for Carbohydrates

Fabian Grünewald[1*], Mats H. Punt[1*], Elizabeth E. Jefferys[2], Petteri A. Vainikka[1], Melanie König[1], Valtteri Virtanen[3], Travis A. Meyer[6], Weria Pezeshkian[1,5], Adam J. Gormley[6], Maarit Karonen[3], Mark S. P. Sansom[2], Paulo C. T Souza[4†], Siewert J. Marrink[1†]

[1] Groningen Biomolecular Sciences and Biotechnology Institute and Zernike Institute for Advanced Materials, University of Groningen, Groningen, The Netherlands

[2] Department of Biochemistry, University of Oxford, South Parks Road, Oxford, United Kingdom, OX1 3QU

[3]Natural Chemistry Research Group, Department of Chemistry, University of Turku, FI-20014 Turku, Finland

[4]Molecular Microbiology and Structural Biochemistry, UMR 5086 CNRS and University of Lyon, Lyon, France

[5]The Niels Bohr International Academy, Niels Bohr Institute, University of Copenhagen, Copenhagen, Denmark

[6]Department of Biomedical Engineering, Rutgers, The State University of New Jersey, Piscataway, NJ, USA

* shared first authors

† corresponding authors

s.j.marrink@rug.nl

paulo.telles-de-souza@ibcp.fr

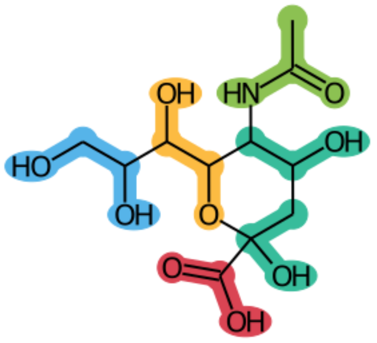

Figure S1. Mapping of Neu5Ac carbohydrate. *Colors correspond to bead types in Figure 1 and Figure 4 of the main paper.*

Table S1. Bond lengths (nm) of α- and β-glucose.

Bond	α-D-GLC	β-D-GLC	%-diff
A - B	0.322	0.330	2.45
A - C	0.389	0.409	5.0
B - C	0.349	0.344	1.4

Table S2. Experimental partitioning coefficients.

Sugar	CODE	Log P	Exp	SD (n=3)	CG_final	CG_SE
D-glucose	GLC	-3.12	-17.81	0.57	-16.32	0.23
D-mannose	MAN	-2.61	-14.9	0.17	-16.03	0.22
D-galactose	GAL	-3.07	-17.52	0.17	-16.16	0.28
N-acetylglucosamine	GlcNAc	-3.03	-17.29	0.34	-16.02	0.33
N-acetylneuraminic acid	NMC	-4.4	-25.11	0.46	-21.39	0.29
D-glucuronic acid	GLA	-3.26	-18.61	0.11	-18.17	0.31
D-fucose	LFUC	-2.26	-12.9	0.34	-11.09	0.23
L-rhamnose	LRHA	-2.26	-12.9	0.23	-11.11	0.24
D-xylose	XYL	-2.43	-13.87	0.11	-13.06	0.19
Inositol	INO	-3.49	-19.92	0.23	-19.42	0.26
Trehalose*	TREH*	-3.77	-21.52	0.29	-20.61	0.42

*value taken from 10.1016/j.carres.2004.12.038

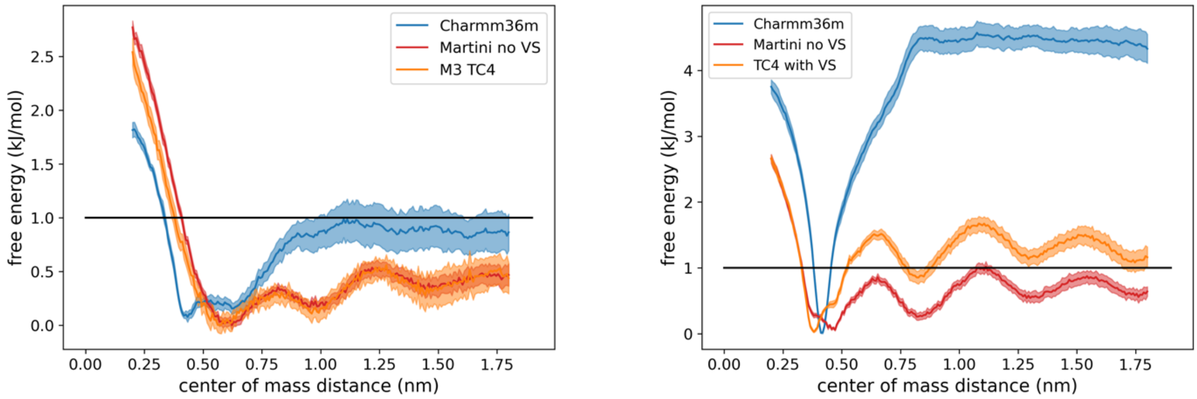

Figure S2. Potential of mean force (PMF) of glucose - indole interaction. *We assessed the effectiveness of the virtual site (VS) by computing the potential of mean force (PMF) profiles between indole and glucose in solution without (left panel) and with orientation restraints (right panel). The orientation restraints enforce a planar interaction. We observe a reasonable agreement between Martini 3 and CHARMM36m, in case of the orientation free PMF. When planarity is enforced, only the Martini model with VS shows an increased binding free energy in agreement with the atomistic simulation. However, the atomistic binding is stronger than what is recovered in Martini. Therefore, on the one hand inclusion of the VS helps in capturing aromatic interactions in general. On the other hand, in situations where a special orientation is enforced as, for example, in a tight binding pocket interactions in Martini could be underestimated.*

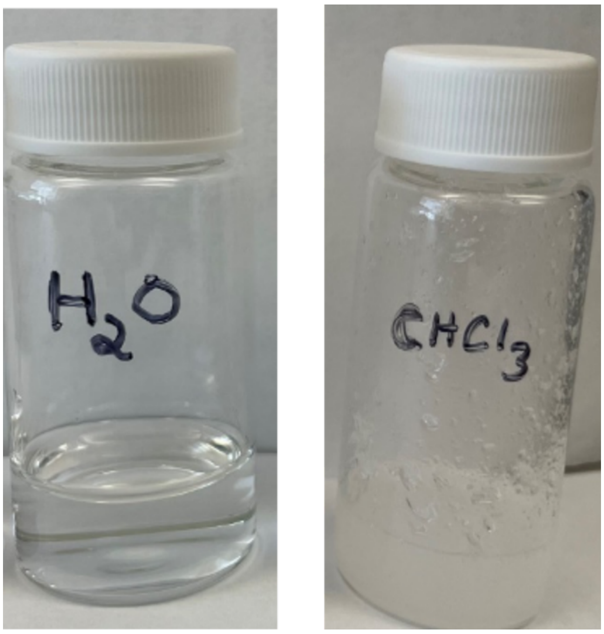

Figure S3. Experimental solubility assessment. *Image taken of 10% w/w Dextran (15-20 kDa) with two solvents (left to right - ultrapure H_2O, and chloroform) after brief vortexing and 1 hour of equilibration at 22°C. Presence of opaque white clumps was taken as indication of insolubility in acetone, toluene, and chloroform.*

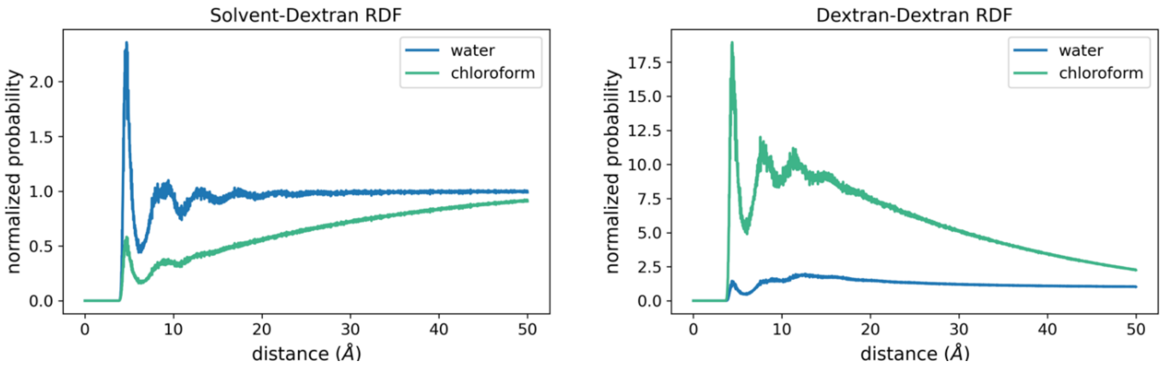

Figure S4. Radial distribution functions of dextran with solvents and itself. *RDFs of the dextran beads with two different solvents, water and chloroform (left panel) were computed from 500 frames of the solution simulations. The RDF between dextran and water shows a pronounced peak at around 0.45nm corresponding to the first solvation shell. In contrast, the green curve corresponding to the RDF between dextran and chloroform shows a peak which is smaller than 1 indicating that the average interactions are unfavorable as expected in a non-solvent. The self RDF between dextran, computed in the same fashion, (right panel) confirms this conclusion. Taken together, these RDFs clearly demonstrate that dextran at this concentration is fully solvated in water whereas it is collapsed in chloroform.*

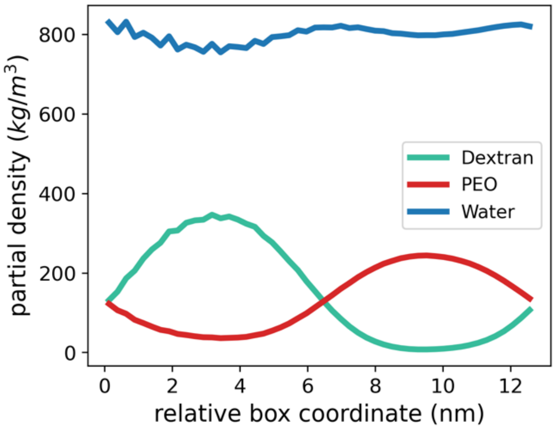

***Figure S5. Partial densities of PEO, dextran and water**. Density profiles are shown of the ATPS of dextran and PEO in water along the z-axis of the simulation box. Clearly dextran is depleted from the PEO phase and vice versa, indicating a phase separation between the two polymers. However, the water has an almost constant partial density across the simulation box, which shows that both polymer phases are still hydrated. This corresponds to the expected density profiles for an ATPS system. We further note that water density in the dextran phase is ever so slightly less than in the PEO phase. This behavior is in qualitative agreement with experimental measurements of partitioning in higher molecular weight dextran PEO systems.*[1–3]

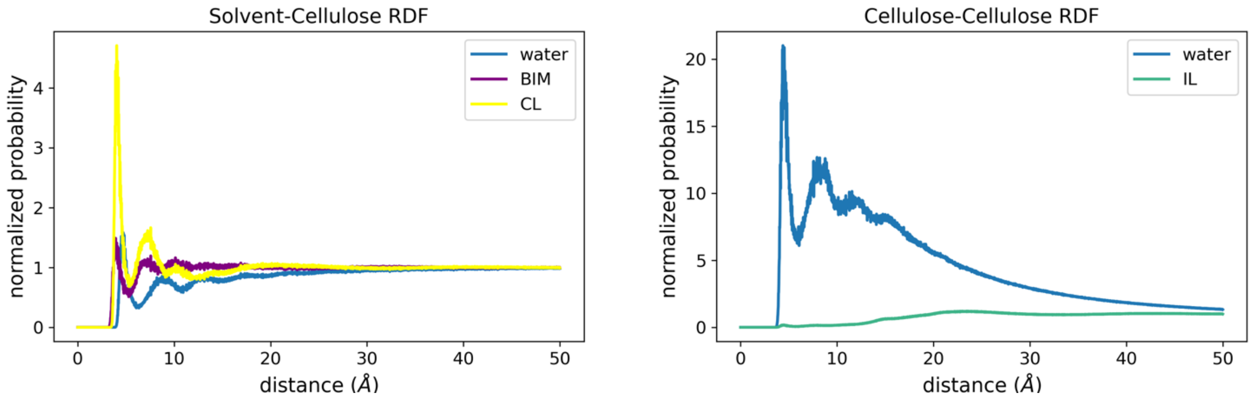

***Figure S6. Radial distribution functions of cellulose with solvents and itself.** RDFs of the cellulose beads with two different solvents (left panel) namely water and the ionic liquid [BMIM][CL] were computed from 500 frames of the solution simulations. The RDF between the CL of the ionic-liquid and cellulose (yellow) shows a pronounced peak at around 4.5Å corresponding to the first solvation shell. In contrast the blue curve corresponding to the RDF between cellulose and water shows a peak in that region, which is much smaller indicating that the average interactions are less favorable as expected in a non-solvent. The self RDF between cellulose, computed in the same fashion, (right panel) confirms this conclusion. Clearly the self-interaction in water (blue) is much higher than in the ionic-liquid (green). Taken together, these RDFs clearly demonstrate that cellulose at this concentration is fully solvated in the ionic liquid whereas it is aggregated in water.*

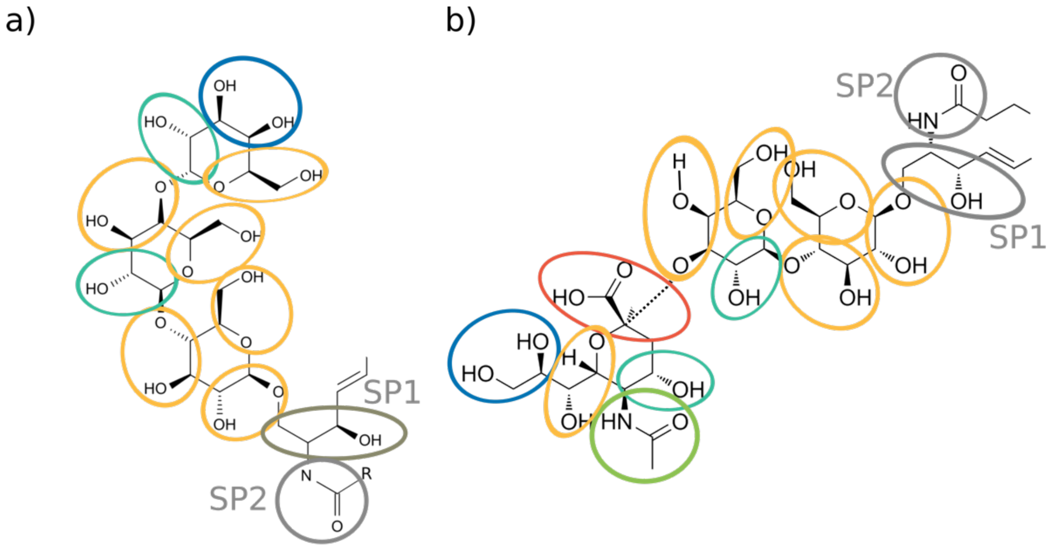

***Figure S7. Mapping of glycolipids**. Gb3 (a) and GM3 (b) with colors of the beads corresponding to Figure 1 and Figure 4 in the main manuscript. Linker beads are mapped as indicated with bead-types SP2 for the amide moiety and SP1 for the alcohol moiety. The rest of the tails is taken from the default Martini 3 paper. Note the TC4 central site has been omitted from the mapping but is part of the parameter set.*

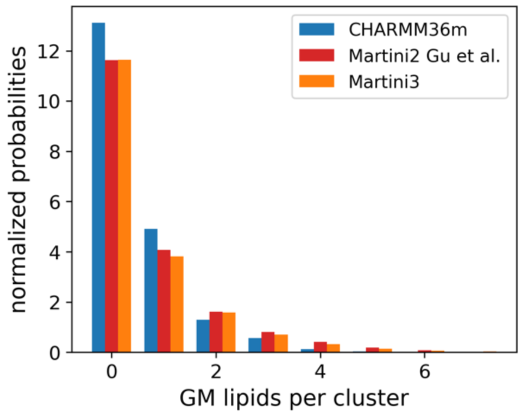

Figure S8. Cluster size distribution for GM3 lipids in CHARMM36m, Martini 2[4] and Martini 3.

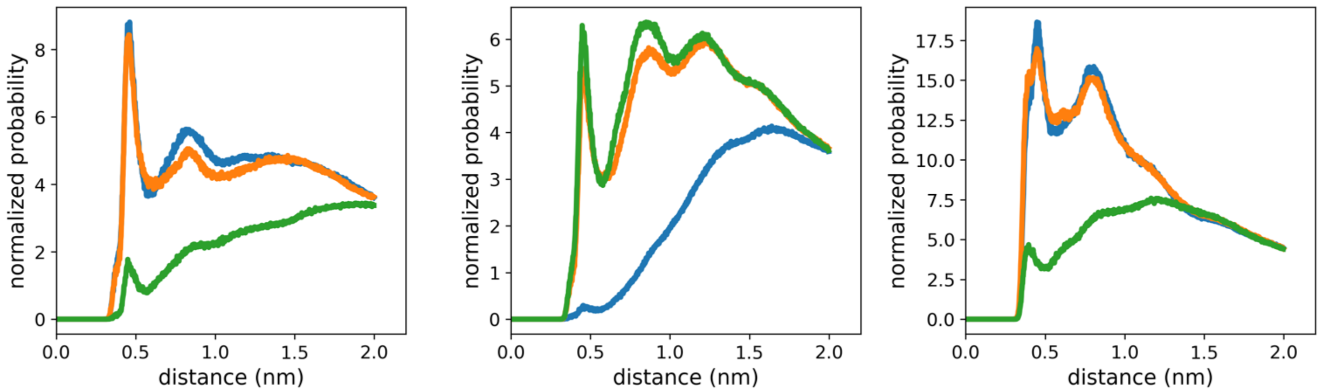

Figure S9. Site specific RDFs of Gb3 with Shiga Toxin binding sites 1-3. *GAL1 (orange) GAL2 (blue) and GLC3 (orange) are sequentially numbered with decreasing distance to the linker. Binding sites were taken as the whole residue at the Martini level as reported from the analysis of the X-ray crystal structure, that is, Table 3 and Table 4 in reference* [5].

Table S3. Force fields used for all-atom simulations of carbohydrates

AA SUGAR	CODE	FF
β-D-glucose	GLC	GLYCAM06h
β-D-mannose	MAN	GLYCAM06h
β-D-galactose	GAL	CHARMM36
β-L-fucose	LFUC	CHARMM36
β-L-rhamnose	LRHA	CHARMM36
β-D-ribofuranose	RIBF	CHARMM36
β-D-xylopyranose	XYL	CHARMM36
β -D-fructofuranose	FRUF	CHARMM36
Inositol	INO	GROMOS54a7 AA
β-D-glucuronic acid	GLA	CHARMM36
β-D-N-acetylglucosamine	GYN	GLYCAM06h
β-D-N-acetylneuraminic acid	NMC	CHARMM36
β-D-glucosamine	GCN	CHARMM36
Lactose (β1,4)	LAC	CHARMM36
Sucrose (α1,2)	SUCR	GROMOS54a7 AA
Trehalose (α1,1)	TREH	CHARMM36

References

(1) Großmann, C.; Tintinger, R.; Zhu, J.; Maurer, G. Partitioning of Low Molecular Combination Peptides in Aqueous Two-Phase Systems of Poly(Ethylene Glycol) and Dextran in the Presence of Small Amounts of K2HPO4/KH2PO4 Buffer at 293 k: Experimental Results and Predictions. *Biotechnol Bioeng* **1998**, *60* (6), 699–711.

(2) Tintinger, R.; Zhu, J.; Grossmann, C.; Maurer, G. Partitioning of Some Amino Acids and Low Molecular Mass Peptides in Aqueous Two-Phase Systems of Poly(Ethylene Glycol) and Dextran in the Presence of Small Amounts of K2HPO4/ KH2PO4-Buffer at 293 K: Experimental Results and Correlation. *J Chem Eng Data* **1997**, *42* (5), 975–984.

(3) Tintinger, R.; Zhu, J.; Grossmann, C.; Maurer, G. Partitioning of Some Amino Acids and Low Molecular Mass Peptides in Aqueous Two-Phase Systems of Poly(Ethylene Glycol) and Dextran in the Presence of Small Amounts of K 2 HPO 4 /KH 2 PO 4 -Buffer at 293 K: Experimental Results and Correlation. *J Chem Eng Data* **1997**, *42* (5), 975–984.

(4) Gu, R.-X.; Ingólfsson, H. I.; de Vries, A. H.; Marrink, S. J.; Tieleman, D. P. Ganglioside-Lipid and Ganglioside-Protein Interactions Revealed by Coarse-Grained and Atomistic Molecular Dynamics Simulations. *J Phys Chem B* **2017**, *121* (15), 3262–3275.

(5) Ling, H.; Boodhoo, A.; Hazes, B.; Cummings, M. D.; Armstrong, G. D.; Brunton, J. L.; Read, R. J. Structure of the Shiga-like Toxin I B-Pentamer Complexed with an Analogue of Its Receptor Gb $_3$ ʾ. *Biochemistry* **1998**, *37* (7), 1777–1788.
